# Narrow-syntax and post-spellout movements: evidence from the syntax of idiomatic expressions in Arabic varieties

**DOI:** 10.1016/j.heliyon.2020.e03658

**Published:** 2020-04-14

**Authors:** Mohammad Rayyan, Marwan Jarrah, Nimer Abusalim

**Affiliations:** School of Foreign Languages, University of Jordan, Jordan

**Keywords:** Arts & humanities, Linguistics, Idiomatic expressions, Arabic varieties, Word order, PF movements, The architecture of language

## Abstract

This research paper investigates the syntax of idiomatic expressions consisting of the verb and the object/accompanying adjunct (VP idiomatic expressions, henceforth) in two Arabic varieties: Modern Standard Arabic (MSA) and Jordanian Arabic (JA). It shows that in order for VP idiomatic expressions to obtain their idiomatic reading, the predominate pattern of the word order in each variety (i.e., the VSO word order in MSA, but the SVO word order in JA) should be used; otherwise idiomaticity is not possible (with few exceptional cases discussed in the paper). We offer evidence that this restriction on the idiomaticity of VP idiomatic expressions in Arabic varieties follows from a proposed condition that the subject (even if it is not part of the idiomatic expression) and the verb (in addition to the object) should maintain a structurally local relation with each other in the narrow syntax, i.e. they should be included in the vP phase before the spell-out point. The paper shows that the movement of the verb to T^0^ in MSA and JA or lack thereof does not break idiomaticity, nor does the movement of the subject to Spec,TP in JA. These facts are taken as an indication that a distinction between narrow-syntax and post-spellout movements should be made. This provides evidence for proposals that distinguish between pre- and post-spellout movements (cf. Chomsky 2001, among others).

## 1 Introduction

Idiomatic expressions are regarded as a significant area of linguistic research, where different theories attempt to use them in favour of their plausibility or, at the same time, against the application or the rationale of competing theories (O'grady, 1998; Ifill, 2003; Bruening, 2010; Espinal and Mateu, 2010; and Larson, 2017; among many others). Such a premium is placed on idiomatic expressions due to their property being non-compositional, i.e. their meaning is not determined through the calculous of their individual parts. This is the reason why idiomaticity is strongly tied to non-compositionality (see Fernando and Carter, 1996; Grant and Bauer, 2004). Furthermore, under most syntactic theories, idiomatic expressions require a special treatment and even demand extra arguments to account for their structure. The challenge set by such expressions help, nonetheless, to reveal deep properties of human language architecture and how various syntactic constructions are produced and perceived (Jackendoff, 1997; Gibbs, 2007).

Contributing to the ongoing research on idiomatic expressions, this paper takes a different approach to investigate idiomatic expressions. It does not embark on any attempt to explore how idiomatic expressions are lexically derived or perceived. Rather, it investigates the word order of such expressions in two Arabic varieties: Modern Standard Arabic (MSA) and Jordanian Arabic (JA).^1^ The selection of these two varieties is not random, as MSA and JA are different with respect to the unmarked (i.e. predominate) word order used. In MSA, the unmarked word order is, as widely assumed in the related literature, the VSO word order (see Bakir, 1980; El-Yasin, 1985; Fassi Fehri, 1993; Jarrah, 2020; among many others), whereas the SVO word order is the predominate word order in JA which shares this property of word order with almost all other Arabic vernaculars (see Aoun et al., 1994 for Lebanese Arabic; Mohammad, 2000 for Palestinian Arabic; Benmamoun, 2000 for Egyptian Arabic; Fassi Fehri, 1993 for Moroccan Arabic; see also Jarrah, 2017, 2019a for more discussion about word order in JA).^2^ However, although the two varieties are different regarding the selection of the predominate word order, they are mutually intelligible. This situation clearly excludes any possibility that the lexicon of each variety affects its syntax or the mechanisms of word order formation.

In this paper, we demonstrate that the formation of the word order of idiomatic expressions consisting of the verb and the object (VP idiomatic expressions, henceforth) in these two varieties fares well under the Minimalist Program that argues for an intermediary procedure (i.e. the computational system of human beings; C_HL_) that connects the lexicon with the interface systems of language which are the Phonetic Form (PF) and the Logical Form (LF) (Chomsky, 1995). VP idiomatic expressions are a direct manifestation of the workings of C_HL_. This does not suggest that MSA and JA have a different C_HL_, but they do not share the same application of syntactic operations that generate word orders on the surface. This gives rise to relatively disparate outcomes whose difference lies in the presence of a particular syntactic operation (i.e. the movement of the subject to Spec,TP in JA and its absence in MSA). This assumption supports the view that C_HL_ should be treated as a necessary component of the language architecture. Additionally, this paper provides evidence for the assumption that head movement and movements triggered only to satisfy the EPP feature on heads occur at PF, not in the narrow syntax (C_HL_), i.e. they occur after the spell-out point. We show that such types of movements only have effects on the surface form of sentences without any discernible impact on the meaning of the relevant sentences in the Arabic grammar.

This paper is structured as follows. Section 2 provides an overview of the Minimalist Program (Chomsky, 1995) with a particular focus on its arguments concerning the position of syntax in the language design. This section also provides the arguments for and against the presence of PF movements (which have no impact on the meaning of sentences). Section 3 presents the main observation the current paper explores; VP idiomatic expressions in JA are only permitted under the SVO word order, whereas VP idiomatic expressions in MSA mostly exhibit the VSO word order. Section 4 provides the syntactic account of this observation, using the Minimalist Program, which is shown to be a sufficient model of analysis that captures the relevant differences between MSA and JA. This section also offers evidence in favour of PF movement. Section 5 concludes the research.

## 2 Theoretical background

### 2.1 The Minimalist Program (MP): an overview

**Figure 1:**
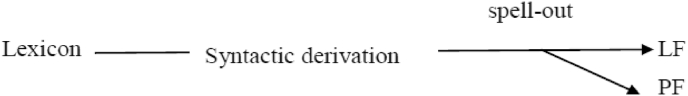
MP's model of sentence formation.

The Minimalist Program (MP), a model of sentence syntactic analysis within the Principles and Parameters approach (Chomsky, 1981, 1986), attempts to explore natural language processing with a special focus on how constituents, phrases, and sentences are computed (Chomsky, 1995, Chomsky, 2000, Chomsky, 2001). It is best viewed as a development over previous syntactic models within Generative Grammar (i.e. the so-called Government and Binding; Chomsky, 1981). The MP eliminates the representational levels of Deep Structure and Surface Structure, which are shown to be theory-internal with no inherently empirical advantage. The MP involves only two levels of representation: the Phonetic Form (PF) and the Logical Form (LF) ''which constitute the interface levels to other cognitive systems (‘bare output conditions’).'' (Fuß, 2005: 24). LF and PF are linked to the lexicon (where lexical items are stored) through the computational system of human beings, i.e. C_HL_, where syntactic operations are applied. Such syntactic operations are said to occur before the spell-out point which the sentence's derivation is transferred to LF and PF levels. This model is represented in Figure 1 (see also Fuß, 2005: 24):

Syntactic operations applied before the spell-out point have both semantic and phonetic impacts on the relevant derivation. On the other hand, any operation that is applied only at LF has no PF consequences. Likewise, any operation that takes place at the PF level of representation has no LF consequences. PF and LF are viewed as separate levels that are not directly connected to each other, as Figure 1 shows. Furthermore, any operation that occurs at LF or PF has no impact on the C_HL_; there are no reversible syntactic operations. In this research and based on evidence from VP idiomatic expressions in MSA and JA, we provide evidence for the presence of C_HL_ and PF movements that have no impact on C_HL._

In the following subsection, we provide more detail about the debate regarding the presence of PF movements, including the so-called head movement.

### 2.2 Movement at PF

Since the inception of the MP, the application of head movement has been controversial. Some have argued that it occurs in the C_HL,_ while others propose that it applies at the PF. For instance, Chomsky (2001) proposes that head movement should be relegated to the phonological component, stating that there is “the possibility that V-raising is […] not part of the narrow-syntactic computation [the C_HL_] but an operation of the phonological component” (Chomsky, 2001: 47). Chomsky (2001) mentions that “[t]here are some reasons to suspect that a substantial core of head raising processes, excluding incorporation in the sense of Baker (1985), may fall within the phonological component” (Chomsky, 2001: 47). Chomsky (2001) suggests the following reasons for why head movement should be relegated from a narrow-syntax process to a PF operation:^3^

i. **No semantic effect**. Since verbs do not receive a different LF interpretation when they undergo movement, unlike Phrasal XP-movement, which does leave considerable LF variations, verb movement is more like phonological movement. A verb remaining in-situ or raising has the same LF interpretation, across languages, suggesting no semantic evidence/motivation for movement, and leaving no scope or reconstruction effects in the process. A known fact about PF-movement is that it also does not leave any semantic effects.
ii. **No motivation for movement**. The trigger for movement does not seem to distinguish between head movement triggers, and phrasal movement triggers. Roberts (2011) illustrates that one example is T^0^ in French, a language where the DP subject moves to Spec,TP and V^0^-movement to T^0^. It is clear that T^0^ must contain the relevant triggers for both movements. The solution could be to ‘enrich’ the system to make such distinctions, a suggestion Chomsky (2001) finds no need for if head-movement was considered not to be within the narrow syntax.
iii. **Moved heads do not c-command their traces**. Chomsky (2001) adds that the moved head cannot c-command its trace if head movement is seen as adjoining one head to another.
iv. **Violation of the Extension Condition**. In the MP, movement may target XPs, in which case, semantic effects may arise. This operation may also apply to a head, i.e. X^0^. However, the problem with head movement is that it targets head positions, meaning that it will violate the Extension Condition which requires that all movements extend the root of the structure that they apply to. The derived structure is thus counter-cyclic (violating the Extension Condition). Chomsky (2001) adds that we will need an explanation for why successive-cyclic movement, which is clearly found for phrasal movement, does not manifest for head movement.

On the other hand, several researchers have argued against the relegation of head movement to PF. For instance, Matushansky (2006) claims that head movement is syntactically similar to any other XP-movement. The two types of movement are a similar phenomenon sharing the same landing site and are triggered by the same factors. For Matushansky (2006), the only difference between XP movement and head movement is in the Morphological-Merger process that is responsible for adjunction of the headed X^0^ and Y^0^ under adjacency, to form one syntactic object. Likewise, Hosono (2018) argues that ‘no semantic effects’ does not equal ‘no narrow syntactic movement’. Hosono mentions that “the constraint that movement can occur only when a new semantic effect occurs on a raised category in its raised position is no longer imposed on movement” (Hosono, 2018: 7). In this research, we provide evidence that head movement as well as subject raising (to Spec,TP) are PF operations that occur after the spell-out point where the C_HL_ is interfaced with PF and LF.

In the following section, we explore word order variation of idiomatic expressions in MSA and JA.

## 3 MSA vs. JA idiomatic expressions

This section shows that VP idiomatic expressions in MSA and JA are different with respect to the word order under which they are permitted. The idiomatic expressions in each variety follow the typical pattern of word order formation. VP idiomatic expressions in MSA should occur in VSO clauses (or SVO clauses as long as the preverbal subject is a topic), while JA idiomatic expressions are highly preferable in SVO clauses. Although this observation looks predictable, it is significant as it constitutes empirical evidence for the presence of narrow-syntax and post-spellout movements in Arabic grammar.

### 3.1 Word order of predicate VP idiomatic expressions in MSA

Examining almost all VP idiomatic expressions in MSA, it is clear that VP idiomatic expressions should occur in clauses with VSO word order, which is widely considered the unmarked (predominate) order in this Arabic variety (see Mohammad, 1990, 2000; Fassi Fehri, 1993, Fassi Fehri, 2012; Aoun et al., 1994; Shlonsky, 1997; Benmamoun, 2000; Aoun et al., 2010; and Jarrah, 2019a). Consider the following examples:^4^^,^^5^

**Table:** 

(1)	ʔibtalaʕa	ʔal-muharribu:n	ʔatˤ-tˤoʕm-a
	swallowed.3SG.M	DEF-smugglers.NOM	DEF-bait-ACC
	Lit. 'The smugglers swallowed the bait.'		
	Idiomatic Reading: 'The smugglers were deceived.'		

Sentence (1) begins with the verb *ʔibtalaʕa* ‘swallowed’, followed immediately by the subject *ʔalmuharribu:n* ‘smugglers’ (which is a referential and not part of the idiomatic expression), then the direct object *ʔaltˤoʕm* 'the bait'. If sentence (1) changes from a VSO word order to an SVO word order, the idiomatic reading of VP holds, as long as the preverbal subject is a topic (see 2a).^6^ If the preverbal subject is a focus, the idiomatic reading of VP disappears (2b,c). (We translate SVO sentences as considering the subject a topic not a subject, following the general assumption that the subject in SVO sentences is a topic, see the discussion below for details).

**Table:** 

(2)	a.	ʔal-muharribu:n	ʔibtalaʕ-u	ʔatˤ-tˤoʕm-a	
		DEF-smugglers.NOM	swallowed-3PL.M	DEF-bait-ACC	
		Lit. 'The smugglers, they swallowed the bait.'			
		Idiomatic Reading: 'The smugglers, they were deceived'.			

	b.	∗muharribu:n	ʔibtalaʕ-u	ʔatˤ-tˤoʕm-a	
		smugglers.NOM	swallowed-3PL.M	DEF-bait-ACC	
		Lit. 'It was smugglers who swallowed the bait.'			
		Idiomatic Reading: Not available			

	c.	∗muharribu:n	(χatˤi:ru:n)	ʔibtalaʕ-u	ʔatˤ-tˤoʕm-a
		smugglers.NOM	dangerous	swallowed-3PL.M	DEF-bait-ACC
		Lit. 'It was dangerous smugglers who swallowed the bait.'			
		Idiomatic Reading: Not available			

Note that a focalized subject does not cancel the idiomatic reading of VP in VSO clauses. This is clearly shown in the following example:

**Table:** 

(3)	ʔibtalaʕ-u	muharribu:n	χatˤi:ru:n	ʔatˤ-tˤoʕm-a
	swallowed-3PL.M	DEF-smugglers.NOM	dangerous	DEF-bait-ACC
	Lit. 'It was dangerous smugglers who swallowed the bait.'			
	Idiomatic Reading: 'It was dangerous smugglers who were deceived.'			

Furthermore, when other word order permutations are used, idiomatic readings are not maintained. This is evidenced in the following examples:

**Table:** 

(4)	a.	ʔatˤ-tˤoʕm-u	ʔal-muharribu:n	ʔibtalaʕ-uh	(OSV)
		DEF-bait-NOM	DEF-smugglers.NOM	swallowed.3PL.M-it	
		Lit. 'The bait, the smugglers swallowed (it).'			
		Idiomatic Reading: Not available			

	b.	ʔatˤ-tˤoʕm-u	ʔibtalaʕa-hu	ʔal-muharribu:n	(OVS)
		DEF-bait-NOM	swallowed.3PL.M-it	DEF-smugglers.NOM	
		Lit. 'The bait, the smugglers swallowed (it).'			
		Idiomatic Reading: Not available			

	c.	ʔibtalaʕ-u	ʔatˤ-tˤoʕm-a	ʔal-muharribu:n	(VOS)
		swallowed-3PL.M	DEF-bait-ACC	DEF-smugglers.NOM	
		Lit. 'The bait, the smugglers swallowed (it).'			
		Idiomatic Reading: Not available			

	d.	ʔal-muharribu:n	ʔatˤ-tˤoʕm-u	ʔibtalaʕ-uh	(SOV)
		DEF-smugglers.NOM	DEF-bait-NOM	swallowed.3PL.M-it	
		Lit. 'The bait, the smugglers swallowed (it).'			
		Idiomatic Reading: Not available			

The examples in (4) clearly show that VP idiomatic expressions are only maintained in MSA in VSO clauses and SVO clauses provided that the subject is a topic; any change to these word orders renders idiomaticity impossible, although the use of other word orders is still a productive process in the MSA grammar. The examples below demonstrate the same observation (we focus here on the VSO-SVO alternation; however, idiomaticity is also broken with other marked word orders).

**Table:** 

(5)	a.	ʕa:da	ʔal-fari:q-u	fa:riɣa	ʔal-jadejn-i	
		returned.3SG.M	DEF-team-NOM	empty	DEF-hands-GEN	
		Lit. 'The team returned empty handed.'				
		Idiomatic reading: 'The team did not achieve the objectives.'				

	b.	∗(ʔal-)fari:q-u	ʕa:da	fa:riɣa	ʔal-jadejn-i	
		DEF-team-NOM	returned.3SG.M	empty	DEF-hands-GEN	
		Lit. 'The team, it returned empty handed.'				
		Idiomatic reading: 'The team, it did not achieve the objectives.'				
		Idiomatic reading: Not available, with the pre-verbal subject interpreted as a focus.				

(6)	a.	kaʃafa	ʔaʃ-ʃa:b-u	ʕan	wadʒh-i-hi	ʔal-ħaqi:q-i
		uncovered.3SG.M	DEF-young man-NOM	about	face-GEN-his	DEF-real-GEN
		Lit. 'The young man uncovered his real face.'				
		Idiomatic reading: 'The young man revealed his true intentions.’				

	b.	∗(ʔaʃ-)ʃa:b-u	kaʃafa	ʕan	wadʒh-i-hi	ʔal-ħaqi:q-i
		DEF-young man-NOM	uncovered.3SG.M	about	face-GEN-his	DEF-real-GEN
		Lit. 'The young man, he uncovered his real face.'				
		Idiomatic reading: 'The young man, he revealed his true intentions.’				
		Idiomatic reading: Not available, with the pre-verbal subject interpreted as a focus.				

The examples in (5–6) show that the MSA idiomatic readings prefer the VSO word order or the SVO word order when the subject is a topic.

According to the main generative literature on the Arabic clause structure of MSA, the VSO word order in this Arabic variety is assumed to be derived through the movement of the verb to the tense affix, i.e. T^0^, that heads TP, whereas the subject remains in situ (in Spec,vP), hence the position of the subject to the right of the tensed verb on the surface (see Benmamoun, 2017 for a general discussion). This analysis is known in the relevant literature as the non-movement hypothesis (Fassi Fehri, 1993; Shlonsky, 1997; Mohammad, 2000; also see Alsarayreh, 2012 for details).^7^ This can be schematically shown in the following tree diagram (throughout the paper, silent copies are inserted between < >; irrelevant details are skipped):

**Figure:**
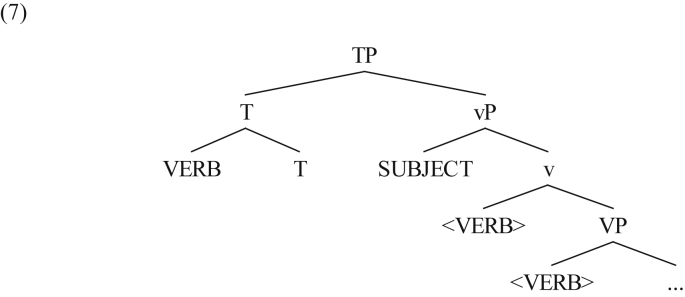

The movement of the verb to T^0^ is argued to be motivated by T^0^'s requirement to be lexically realized (see Benmamoun, 2000; Baker, 2003; Jarrah, 2017). Empirical evidence that supports this hypothesis comes from the fact that when the past tense copula *ka:n* is used, the verb normally appears to the right of the subject, as shown in the following sentence:^8^

**Table:** 

(8)	ka:n	ʔal-walad-u	jalʕabu	bi-l-kurat-a
	was.3SG.M	DEF-boy-NOM	playing	with-DEF-ball-GEN
	‘The boy, he was playing with the ball.’			

With the assumption that *ka:n* is adjoined to the tense affix, there is no need for the verb to move T^0^. This can be schematically shown as follows.

**Figure:**
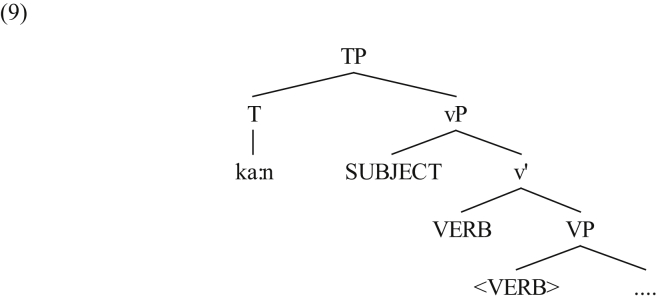

The presence of *ka:n* on T^0^ results in the main verb occurring to the right of the subject; the verb is adjoined to the phonologically-null v^0^ head (Fassi Fehri, 1993, Fassi Fehri, 2012; Baker, 2003; Benmamoun, 2017).

A point that bears mentioning here is that idiomatic readings survive the presence of *ka:n*. With the presence of *ka:n,* while the main verb occurs to the right of the subject, the idiomatic reading still holds in MSA, as shown in the following examples.

**Table:** 

(10)	a.	ʔal-fariq-u	ka:n	jaʕu:du	fa:riɣa	ʔal-jadejn-i
		DEF-team-NOM	was.3SG.M	returned.3SG.M	empty	DEF-hands-GEN
		Lit. 'The team, it was returning empty handed.'				
		Idiomatic reading: 'The team, it was not achieving the objectives.'				

	b.	ka:n	ʔaʃ-ʃa:b-u	jakʃifu		
		was.3SG.M	DEF-young man-NOM	uncovering		
		ʕan	wadʒh-i-hi	ʔal-ħaqi:q-i		
		about	face-GEN-his	DEF-real-GEN		
		Lit. 'The young man, he was uncovering his real face.'				
		Idiomatic reading: 'The young man, he was revealing his true intentions.’				

The bottom line is that the position of the subject relative to the lexical verb is not a necessary condition to obtain idiomaticity in MSA. The subject can appear to the left of the lexical verb as long as T^0^ is lexically supported by *ka:n*. VP idiomatic expressions as such are not one chunk whose lexical integrity is preserved. Rather they are subject to the syntactic processes that are operative in the given language.

In the following subsection, we provide additional evidence for this assumption from JA where the unmarked word order is SVO rather than the VSO.

### 3.2 Word order in predicate idiomatic expressions in JA

Jordanian Arabic (JA) is an Arabic dialect with unmarked SVO word order (El-Yasin, 1985; Abusalim, 2016; Jarrah, 2017, 2020). The VSO word order is only used to express a certain pragmatic/discourse effect. VP idiomatic expressions in JA occur in SVO clauses, as shown in the following example.

**Table:** 

(11)	ʔiz-zalameh	ʔakal	zift
	DEF-man	ate.3SG.M	asphalt
	Lit. 'The man ate asphalt.'		
	Idiomatic reading: 'The man was in a serious problem.''		

If the word order between the subject and the verb is switched, idiomaticity disappears:

**Table:** 

(12)	ʔakal	ʔiz-zalameh	zift
	ate.3SG.M	DEF-man	asphalt
	Lit. 'The man ate asphalt.'		
	Idiomatic reading: Not available		

As is the case in MSA, when other word order permutations are used, the idiomatic reading of VP disappears, as shown in the following examples.

**Table:** 

(13)	a.	zift	ʔiz-zalameh	ʔakal-uh	(OSV)
		asphalt	DEF-man	ate.3SG.M-it	
		Lit. 'Asphalt, the man ate it.'			
		Idiomatic reading: Not available			

	b.	zift	ʔakal-uh	ʔiz-zalameh	(OVS)
		asphalt	ate.3SG.M-it	DEF-man	
		Lit. 'Asphalt, the man ate it.'			
		Idiomatic reading: Not available			

	c.	ʔiz-zalameh	zift	ʔakal-uh	(SOV)
		DEF-man	asphalt	ate.3SG.M-it	
		Lit. 'The man, he ate asphalt.'			
		Idiomatic reading: Not available			

	d.	ʔakal	zift	ʔiz-zalameh	(VOS)
		ate.3SG.M	asphalt	DEF-man	
		Lit. 'Asphalt, the man ate it.'			
		Idiomatic reading: Not available			

The following examples manifest the same observation (again we focus on SVO-VSO alternations).

**Table:** 

(14)	a.	ʔil-mudir	ħaka	min	ra:s	manaxi:r-uh
		DEF-manager	spoke.3SG.M	from	head	noses.his
		Lit. 'The manager spoke from the top of his noses.'				
		Idiomatic reading: 'The manager was arrogant.'				

	b.	ħaka	ʔil-mudir	min	ra:s	manaxi:r-uh
		spoke.3SG.M	DEF-manager	from	head	noses.his
		Lit. 'The manager spoke from the top of his noses.'				
		Idiomatic reading: Not available				

(15)	a.	Sarah	ħatˤtˤ-at	ʔi:d-ha	ʕala:	ʔidʒ-dʒuruħ
		Sarah	put-3SG.F	hand-her	on	DEF-sore
		Lit. 'Sarah out her hand on the sore.'				
		Idiomatic reading: 'Sarah knew the exact problem.'				

	a.	ħatˤtˤ-at	Sarah	ʔi:d-ha	ʕala:	ʔidʒ-dʒuruħ
		put-3SG.F	Sarah	hand-her	on	DEF-sore
		Lit. 'Sarah put her hand on the sore.'				
		Idiomatic reading: Not available.				

(16)	a.	ʕabid	ʔaʕtˤa:-ni	raas	ʔil-χeetˤ	
		Abed	gave.3SG.M-m	tip	DEF-thread	
		Lit. 'Abed gave me the tip of the thread.'				
		Idiomatic reading: 'Abed gave me a hint.'				

	b.	ʔaʕtˤa:-ni	ʕabid	raas	ʔil-χeetˤ	
		gave.3SG.M-m	Abed	tip	DEF-thread	
		Lit. 'Abed gave me the tip of the thread.'				
		Idiomatic reading: 'not available.'				

The examples in (14–16) show that idiomaticity in JA holds when the word order is SVO; when the VSO word order is used, idiomatic readings are deemed not available by our JA informants.

It has been proposed that the subject in JA raises to Spec,TP due to the strong EPP feature in this language (see Jarrah, 2017 for motivation and analysis). This proposal is supported by the observation that the preverbal subject in JA should not be accompanied by a certain informational value (unlike the case in MSA where the subject in SVO clauses should be definite or accompanied with a contrastive intonation; see Moutaouakil, 1989); the subject in JA might be an indefinite, nonspecific entity (Jarrah, 2019b). As for the verb, it has been proposed that the verb moves to adjoin T^0^ as is the case in MSA (Jarrah, 2019b). This is schematically shown in the following tree diagram.

**Figure:**
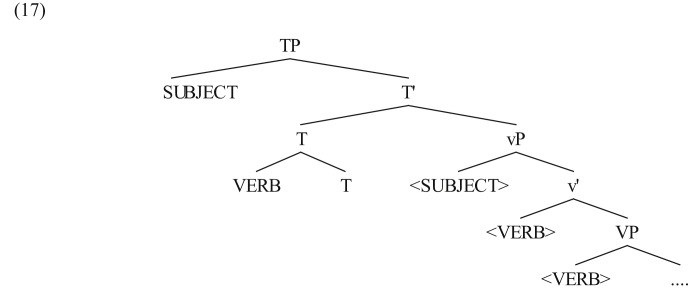

The movement of the subject to Sepc,Tp and the verb to T^0^ result in a situation where the subject precedes the verb on the surface, hence yielding the SVO word order.

When the past tense copula *ka:n* is used in JA, the subject still appears sentence initially, given its movement to Spec,TP. The presence of *ka:n* does not cancel idiomatic readings of VP, as shown in the following examples.

**Table:** 

(18)	a.	ʔiz-zalameh	ka:n	jokil	zift		
		DEF-man	was.SG.M	eating	asphalt		
		Lit. 'The man was eating asphalt.'					
		Idiomatic reading: 'The man was in a serious problem.'					

	b.	ʔil-mudir	ka:n	ħaka	min	ra:s	manaxi:r-uh
		DEF-manager	was.SG.M	speaking	from	head	noses.his
		Lit. 'The manager was speaking from the top of his noses.'					
		Idiomatic reading: 'The manager was arrogant.'					

	c.	Sarah	ka:n-t	ʔitħutˤ			
		Sarah	was-3SG.F	put.3SG.F			
		ʔi:d-ha	ʕala:	ʔidʒ-dʒuruħ			
		hand-her	on	DEF-sore			
		Lit. 'Sarah was putting her hand on the sore.'					
		Idiomatic reading: 'Sarah knew the exact problem.'					

	d.	ʕabid	ka:n	jiʕtˤini	raas	ʔil-χeetˤ	
		Abed	was-3SG.M	gave.3SG.M-me	tip	DEF-thread	
		Lit. 'Abed was giving me the tip of the thread.'					
		Idiomatic reading: 'Abed was giving me a hint.'					

In the examples in (18), the verb is believed not to raise to T^0^ as the latter is lexically supported by *ka:n* (see Jarrah, 2017, 2019b).

Given this background, we reach the following situation: idiomaticity holds in MSA when the word order is VSO, which is the unmarked word order in this language. When T^0^ is lexically supported, idiomaticity still obtains even if the verb appears to the right of the subject. On the other hand, when the subject appears sentence-initially, no idiomaticity generally obtains, only the subject is interpreted as a topic. As for JA, idiomaticity obtains when the subject appears sentence-initially (i.e. SVO); the presence of the verb to the left of the subject forces the literal reading of the sentence. The presence of *ka:n* does not affect idiomaticity. Given this, several questions arise. Why does the presence of a non-topical subject to the left of the tensed verb force the literal reading of the sentence in MSA? Why does this not work when the subject appears to the left of the non-tensed verb (i.e. when T^0^ is lexically supported by *ka:n*) in MSA? Why does the presence of the tensed verb to the left of the subject work against idiomaticity in JA? Table 1 summarizes the main differences between MSA and JA with respect to idiom formation and the use of the word order.

**Table 1:** JA vs. MSA w.r.t. formation of idiomatic expressions.

Variety	Word order	Idiomatic reading
MSA	VSO	√
	SVO	Only when the subject is topical
	SOV; OVS; OSV; VOS	X
JA	VSO	X
	SVO	√
	SOV; OVS; OSV; VOS	X

In the following section, we provide our account of these data, arguing that there should be two types of movement: narrow-syntax movement and post-spellout movement. This essentially supports the view that syntax is an independent ingredient of the language design which also includes post-syntax movements with effects only on surface.

## 4 Narrow syntax vs. PF

As for MSA, we propose that although the subject is not part of the VP idiomatic expression, it should have a certain syntactic (i.e., configurational) relation with the verb and the object in order to make the VP idiomatic. Our proposal is that the subject and the verb should stay in the same vP phase in the narrow syntax. If one of them undergoes some syntactic movement (i.e. XP-movement), it breaks up this local relation, something that makes idiomaticity not available. When the word order used is VSO, the subject remains in Spec,vP, while the verb adjoins to T^0^. At face value, this would count as a violation of our proposed condition on the availability of VP idiomatic expressions. That is because the verb moves to adjoin to T^0^, hence leaving the maximal projection that originally contains both of them, i.e. vP. On the other hand, with the assumption that head movement is not a narrow syntax process but a post-syntactic movement that is confined to PF (Chomsky, 2000; see also Sigurðsson, 2006), movement of the verb to T^0^ does not have any impact on the local relation between the subject and the verb, if we suppose that this local relation between the verb the subject holds in the narrow syntax.

As for the SVO word order in MSA, we have shown that it only gives rise to VP idiomatic readings when the subject is a topic, but not a focus. It has widely been argued in the related literature that when the subject in SVO clauses of MSA is a topic, it is located in Spec,Topic Phrase (cf. Rizzi, 1997) rather being a true subject that is located in Spec,TP (Mohammad, 2000; Akkal and Gonegai, 2000). The topicalized subject is directly base-generated in the left periphery, while the position of the thematic subject in Spec,vP is filled with a pro (Ouhalla, 1997). In such cases, the verb maintains a local relation with the unpronounced subject, i.e. pro, the reason why SVO clauses may give rise to VP idiomatic readings. The sentence derivation when the subject is a topic is SVO word order, as is schematically represented in (19).^9^

**Figure:**
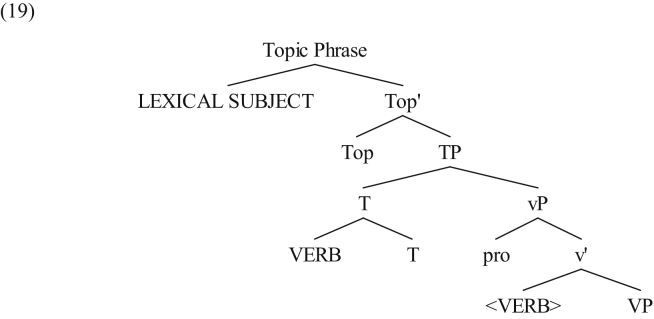

On the other hand, when the subject in SVO clauses is interpreted a focus, it is base-generated in its thematic position, then it moves to the CP domain. This is schematically shown in (20).

**Figure:**
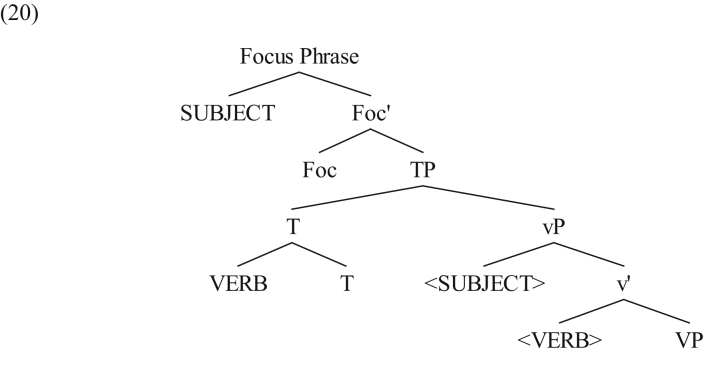

Here the subject undergoes an XP movement which is a narrow-syntax process, hence breaking up the local relation with the verb.

As for cases where T^0^ is lexically supported, it can be suggested that the verb remains in situ and the subject is located in Spec,vP, the ideal environment where idiomatic expressions are formed.

Following this path of analysis, we could account for why idiomatic expressions are not available under other possible permutations of word order in MSA, even if such word orders are possible. In these word orders, the subject, the verb and the object do not share a structurally local domain. For instance, in OVS sentences, the object moves to/is base-generated in the left periphery of the relevant clause (Ouhalla, 1997; Shlonsky, 2000), hence, breaking the structurally local environment where idiomatic expressions are formed.

As for JA, the subject in SVO clauses moves to Spec,TP, something that might be counted as a violation of the syntactic condition of the formation of idiomatic expressions (i.e. the subject and the verb should maintain a structurally local relation with each other in the narrow syntax). However, no violation incurs if we assume that movement to Spec,TP should occur post-syntax as it is mainly motivated by EPP purposes. One direct piece of evidence that the movement of the subject to Spec,TP is not in the narrow syntax comes from the fact that expletives which have no semantic value are used to fill Spec,TP in some languages, including English (Chomsky, 1995). The insertion of expletives in Spec,TP should not be implemented before the spell-out point, otherwise such elements will not be interpretable at LF, causing the derivation to crash. According to Chomsky (1995),

The principle of FI [Full Interpretation] is assumed as a matter of course in phonology; if a symbol in a representation has no sensorimotor interpretation, the representation does not qualify as a PF representation. This is what we called the "interface condition". The same condition applied to LF also entails that every element of the representation have) a (language independent) interpretation (Chomsky, 1995: 27).

Expletives which are elements free of semantically interpretable features would cause the derivation to crash if they were inserted before the spell-out point. With this being the case, the subject and the verb remain too local in the narrow syntax, where the surface position of the subject is ascribed to an operation outside narrow syntax.

For VSO clauses, we follow here Jarrah and Abusalim's (2020) recent proposal that the subject in VSO clauses in JA moves to a position in the low IP area between T^0^ and v^0^ (i.e. Spec,Topic Phrase or Spec,Focus Phrase of the low IP). This movement, as it occurs before the spell-out point, works against the environment that is necessary to obtain VP idiomatic readings in Arabic.

Regarding the cases when T^0^ is lexically supported by *ka:n*, the verb here remains in situ and the subject is still in Spec,vP before the spell-out point. The subject and the verb obtain a local domain before the spell-out point. The following table summarises the main movements and their types in MSA and JA.

Table 2 shows that idiomatic expressions of VP are not maintained in SVO clauses of MSA when the subject is a focus because of the movement of the subject to the CP domain; while they are not obtained in VSO clauses of JA because of the movement of the verb to the left periphery. This discussion brings evidence in favour of the figure in (1), where syntax is viewed as an independent component of the language design. It also brings evidence for the presence of PF movement that has no impact on LF interpretation.

**Table 2:** Movement and idiom formation in MSA and JA.

Variety	Word order	Idiomatic reading	PF movement	C_HL_ movement of the verb	C_HL_ movement of the subject
MSA	VSO	√	√	X	X
	SVO S = topic	√	X	X	X
	SVO S = focus	X	X	X	√
JA	VSO	X	X	√	X
	SVO	√	√	X	X

## 5 Conclusion

This discussion is best fitted under the proposal where a distinction between narrow syntax movement and post-syntax movement is made. The MP provides us with an elegant account of the formation of VP idiomatic expressions in the Arabic variety, providing empirical evidence for the presence of movement, which is shown not to be homogenous. Furthermore, facts on Arabic VP idiomatic expressions make available empirical evidence against any approach that ascribes to the differences between languages (or dialects) to some lexical operations (see simpler syntax; Culicover and Jackendoff, 2005). That is because JA and MSA share the same lexicon to a large extent. MSA is comprehensible to JA speakers. It is difficult to account for the reason for such lexical operations between two varieties whose lexicon is approximately similar.

## Declarations

### Author contribution statement

M. Rayyan and M. Jarrah: Conceived and designed the experiments; Analyzed and interpreted the data; Contributed reagents, materials, analysis tools or data; Wrote the paper.

N. Abu Salim: Performed the experiments.

### Funding statement

This research did not receive any specific grant from funding agencies in the public, commercial, or not-for-profit sectors.

### Competing interest statement

The authors declare no conflict of interest.

### Additional information

No additional information is available for this paper.

## Footnotes

1 The JA examples cited in this paper come from a JA subdialect that is spoken in northern Jordan.

2 Although the topic whether Arabic varieties (including MSA and Arabic dialects) are VSO or SVO languages is controversial (see, e.g., Peled, 2008; Maamouri et al., 2006), there is a general acceptance among researchers that MSA is a VSO language, whereas Arabic dialects are SVO languages (see Parkinson, 1981). In order to examine the widely-held assumption that MSA is a VSO word order, we randomly collect a 100000-word corpus from different areas where MSA is used (i.e. newspapers, books, speeches, etc.) and determine the word order of their sentences. It turns out that 67% of all sentences use a VSO word order, whereas 24% use a SVO word order, a result demonstrating the clear dominance of VSO over other words orders used in this language (other word order permutations stand for 9% of the whole corpus). See Al-Shawashreh (2016) for corpus-based evidence that JA is predominately an SVO language.

3 See Platzack (2013) for more discussion of the phonological view of head movement.

4 We used The Leipzig glossing conventions for the gloss of all Arabic examples mentioned in the text.

5 In MSA, the verb agrees with its subject in Person, Number, and Gender in an SVO clause, whereas in a VSO clause it only agrees with its subject in Person and Gender. This is why the verb in (2) is attached to a 3PL.M morpheme. See Mohammad (1990, 2000), Fassi Fehri (1993), Aoun et al. (1994), Shlonsky (1997); Benmamoun (2000), Aoun et al. (2010), and Jarrah (2020) for proposals and discussions.

6 Note here that the use of a modal/hypothetical particle does not cancel idiomaticity, as long as the right word order is used. This is evidenced in the following examples:

**Table:**

(i)	law	ʔibtalaʕa	ʔal-muharribu:n	ʔatˤ-tˤoʕm-a	
	if	swallowed.3SG.M	DEF-smugglers.NOM	DEF-bait-ACC	
	Lit. 'If the smugglers swallowed the bait….'				
	Idiomatic Reading: 'If the smugglers were deceived….'				
(ii)	min-ʔalmumkin	ʔanna	jabtaliʕa	ʔal-muharribu:n	ʔatˤ-tˤoʕm-a
	possible	if	swallow.IMPRF.3SG.M	DEF-smugglers.NOM	DEF-bait-ACC
	Lit. 'The smugglers might swallow the bait.'				
	Idiomatic Reading: 'The smugglers might be deceived.'				

7 There is another hypothesis (movement hypothesis) where the subject is assumed to move to Spec,TP, while the tensed verb moves to a higher functional phrase (Aoun et al., 1994, 2010). However, this hypothesis suffers from the lack of sufficient empirical evidence, e.g., it is unclear why the verb should move to a higher position (in the CP domain).

8 An anonymous reviewer raised the point that ''using the particle *ka:n* does not fit the main objective for many reasons: (i) this particle is ∗typically∗ inserted before a nominative sentence; however, the examples in the paper do not consistently follow this pattern. (ii) This particle also constraints/controls so many other structures in the same way. (iii) The function of *ka:n* in MSA is completely different from that of JA as it has undergone several (probably 5) synchronic and diachronic grammaticalisation stages, by which it changes its functions and syntactic structure.'' Our rsponce to this point is the following. Actually, *ka:n* is much in Arabic syntax as a diagnostic of verb movement and the structural position of the subject in many Arabic dialects (see Alqassas, 2015; Ouali, 2014, among many others). Additionally, it is not restrictive to use *ka:n* only before a nominative sentence (which we understand here as a nominal sentence): consider the following sentence:

**Table:**

(i)	ka:n	jirsil	ju:suf	ʔil-ʔime:l
	was	sending	Yousef	DEF-email
	'Yousef was sending the email.'			As for the grammaticalization of *ka:n* in JA, actually there is no single study, to the best of our knowledge, has handled this topic. Additionally, the grammaticalization of *ka:n* in JA is hard to pin point as we have no access to any diachronic data. Furthermore, according to main literature in the field, *ka:n* obtains a similar behaviour in MSA and Arabic varieties, including JA (see Alotaibi, 2019 for a recent discussion and overall analysis).

9 We leave the question of why the silent copies of moved elements do not save idiomatic readings open for further research.
